# A Review of Plant-Derived Diterpenoid Biosynthesis: From Structural Scaffold Diversity and Lineage-Associated Distribution to Enzyme Mining and Discovery Strategies

**DOI:** 10.3390/molecules31152653

**Published:** 2026-07-30

**Authors:** Yalan Zhao, Mengyao Li, Shasha Zuo, Xiulin Han, Yupeng Liang

**Affiliations:** 1 Department of Basic Courses, Zunyi Medical and Pharmaceutical College, Zunyi 563006, China; 2 Key Laboratory of Microbial Diversity in Southwest China, Ministry of Education, Yunnan Institute of Microbiology, School of Life Sciences, Yunnan University, Kunming 650500, China; 3 Department of Pharmacy, Zunyi Medical and Pharmaceutical College, Zunyi 563006, China; limyao2020@163.com; 4 School of Basic Medical Sciences, Medicine & Technology College of Zunyi Medical University, Zunyi 563000, China; 15120297265@163.com; 5 Academy for Advanced Interdisciplinary Studies, Nankai University, Tianjin 300350, China

**Keywords:** plant diterpenoids, diterpene biosynthesis, diterpene scaffold, diterpene synthases, cytochrome P450 monooxygenases

## Abstract

Plant diterpenoids are a diverse class of natural products with important ecological roles and wide applications in the pharmaceutical, agricultural, food additive, and chemical industries. Biosynthesis represents a primary strategy for accessing these valuable compounds. However, the identification of downstream tailoring enzymes (hereafter referred to as tailoring enzymes) involved in diterpenoid biosynthetic pathways remains a major bottleneck, particularly in non-model plant species with limited genomic resources. This review summarizes current strategies for discovering plant diterpenoid biosynthetic pathways and recent advances in elucidating their metabolic routes. We further highlight the lineage-biased distribution of diterpene scaffolds across plant taxa. We propose that scaffold enrichment in specific evolutionary lineages, when integrated with enzyme family expansion and functional divergence, may provide a complementary framework for prioritizing candidate tailoring enzymes. Importantly, scaffold enrichment alone cannot establish enzyme function or evolutionary causality; rather, it provides a complementary layer of evidence that can guide future experimental investigation. Future perspectives include predictive substrate–enzyme mapping, computational and generative design of cytochrome P450 enzymes, and the integration of enzyme discovery, structural modeling, and heterologous chassis engineering.

## 1. Introduction

**Figure 1:**
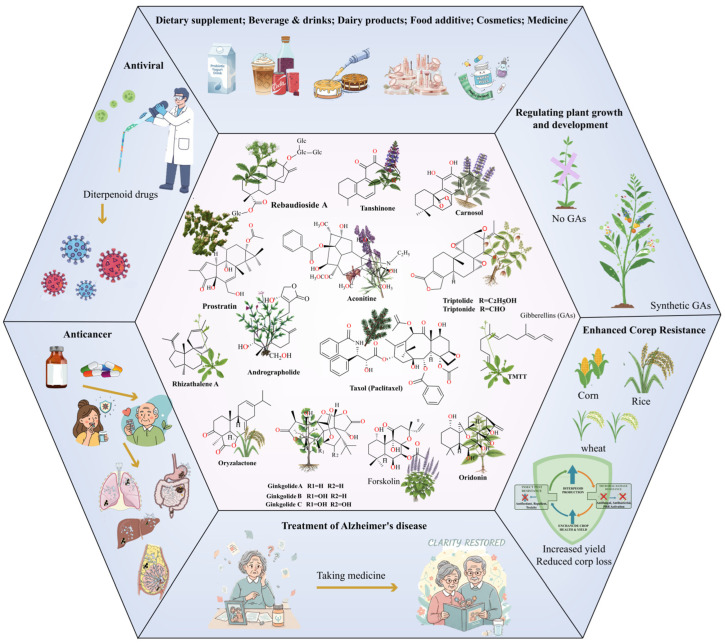
Major plant-derived diterpenoids and their ecological functions and applications.

Diterpenoids (C20) are a major class of plant specialized metabolites and represent an important component of plant chemical diversity. More than 30,000 natural diterpenoids have been reported to date [1], some of which have important applications in medicine, food additives, and the fragrance industry (Figure 1). Diterpenoids often possess highly complex structures and multiple chiral centers. According to the degree of cyclization of their carbon scaffolds, they can be broadly classified into acyclic, bicyclic, tricyclic, tetracyclic, and macrocyclic types [2]. Such structural diversity underlies the wide range of biological activities of diterpenoids, but also makes their biosynthetic pathways difficult to elucidate. Paclitaxel provides a representative example: its complete biosynthetic pathway has long been considered a major challenge in natural product biosynthesis, and key advances have only recently been achieved after decades of research [3].

The chemical diversity of diterpenoids is generated through a modular biosynthetic logic. Diterpene synthases (diTPSs) first cyclize the universal precursor geranylgeranyl diphosphate (GGPP) into a relatively limited set of core carbon scaffolds [4], such as kaurane- and clerodane-type scaffolds. Although more than 60,000 diterpenoids conforming to the C20 rule have been described, they are derived from fewer than 1000 basic scaffold types [1]. The expansion from a limited scaffold repertoire to a large number of specialized metabolites is largely driven by multi-site and sequential modifications of diterpene scaffolds by downstream tailoring enzymes, particularly cytochrome P450 monooxygenases (P450s). Therefore, in several experimentally characterized diterpenoid pathways, expansion and functional divergence of P450 families have been shown to contribute to chemical diversification by generating lineage-specific oxidative modification capacities. However, whether similar evolutionary patterns broadly apply across plant lineages remains unclear [5].

Although the general biosynthetic model of core scaffold formation followed by downstream modification is increasingly clear, complete elucidation of individual diterpenoid pathways remains challenging. To date, systematic characterization of plant diterpenoid pathways remains concentrated on a limited number of representative compounds and compound classes, including paclitaxel [3], tanshinones [6,7,8,9,10], forskolin [11], andrographolide [12,13], ginkgolides [14], casbene [15], and steviol glycosides [16]. For most diterpenoids, especially with respect to functional annotation of downstream modifying enzymes, two major limitations remain. First, many diterpenoids are produced by non-model plants, for which high-quality genomes, multi-omics datasets, and stable genetic transformation systems are often unavailable. This restricts metabolic gene cluster mining and integrated multi-omics screening. Second, P450s constitute a large enzyme family with broad substrate adaptability and extensive functional redundancy. Tissue-specific expression, induced expression, or co-expression patterns alone are often insufficient to accurately identify the key P450s responsible for pathway-specific modifications from large candidate pools [17,18]. Improving the prioritization of candidate tailoring enzymes has therefore become a central bottleneck in the elucidation of plant diterpenoid biosynthetic pathways. We further highlight the lineage-biased distribution of diterpene scaffolds across plant taxa and propose a metabolite-centered, evolution-guided framework for candidate enzyme prioritization. In contrast to conventional gene-centered workflows, this framework starts from scaffold enrichment in specific plant lineages and integrates this information with gene family expansion and functional divergence to narrow candidate enzyme pools.

This bottleneck is closely associated with the ecological and adaptive evolution of plant specialized metabolites (PSMs). As sessile organisms, plants have evolved chemical defense systems centered on PSMs to respond to diverse biotic and abiotic stresses [19]. Diterpenoid accumulation is often inducible, tissue-specific, and environmentally dependent. For example, the biosynthesis of 17-hydroxygeranyllinalool diterpene glycosides (17-HGL-DTGs) in *Nicotiana attenuata* directly mediates resistance to the tobacco hornworm, *Manduca sexta* [20]. Monocots can specifically produce diterpenoid phytoalexins upon pathogen infection [21], while drought stress can reshape the terpenoid metabolic network in *Pinus elliottii* [22]. These examples indicate that diterpenoid metabolism is not uniformly distributed across the plant kingdom but is shaped by lineage evolution, niche adaptation, and environmental selection pressures. Accordingly, enzyme families involved in diterpenoid post-scaffold modification may have undergone gene duplication, expansion, and functional divergence in certain plant lineages, thereby contributing to lineage-specific metabolic capacities.

Compared with highly specialized terminal diterpenoid derivatives, which often have narrow taxonomic distributions, diterpene core scaffolds are relatively more stable across plants and often show enrichment patterns in particular lineages. Thus, core scaffolds not only provide a basis for structural classification, but also offer useful clues for tracing the evolution of associated biosynthetic genes. In this review, we systematically summarize the structural diversity of plant-derived diterpenoids, the lineage distribution of their core scaffolds, and recent progress in the elucidation of representative diterpenoid biosynthetic pathways. Building on this synthesis, we further propose a candidate enzyme discovery strategy that integrates plant chemotaxonomy with molecular phylogenetics. The enrichment of particular diterpene scaffolds in specific plant lineages can provide an initial prioritization signal. This signal can then be integrated with evidence of lineage-specific expansion and functional divergence in tailoring enzyme families derived from multispecies genomic or transcriptomic datasets. We clarify that this metabolite-centered, evolution-guided framework is intended to complement, rather than replace, established gene-centered approaches such as gene cluster mining, co-expression analysis, and homology-based prediction. By integrating lineage-associated scaffold enrichment with enzyme family expansion and functional divergence, it provides an additional layer of evidence for generating testable hypotheses and prioritizing candidate genes within large tailoring enzyme families, particularly P450s, before biochemical and genetic validation.

## 2. Current Strategies for Identifying Plant-Derived Diterpenoid Biosynthetic Pathways

**Figure 2:**
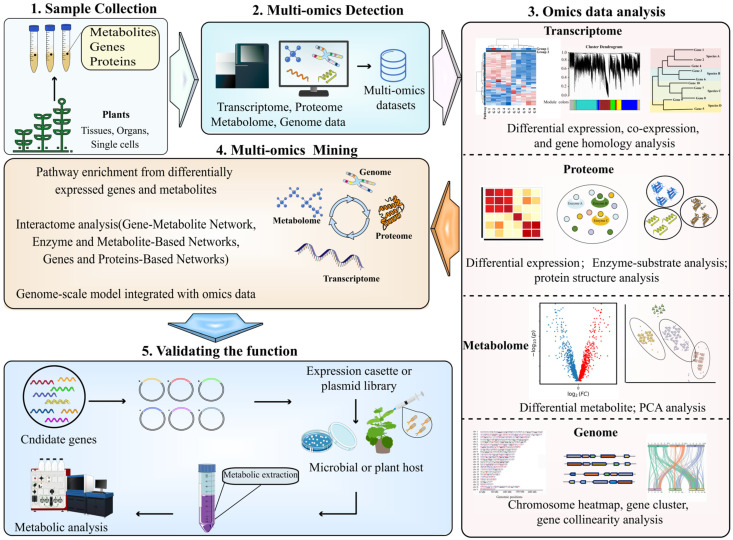
Schematic workflow of multi-omics approaches for the discovery and functional validation of diterpenoid biosynthetic genes.

Multi-omics datasets, including genomics, transcriptomics, metabolomics, and proteomics, have become an important foundation for elucidating plant specialized metabolic pathways [23,24]. For plant-derived diterpenoids, the central challenge is not only to identify diTPSs responsible for scaffold formation, but also to select the key enzymes that truly participate in downstream oxidation, glycosylation, acylation, or rearrangement reactions from large and functionally complex families of modifying enzymes. To address this challenge, current pathway-discovery studies mainly use metabolic gene cluster mining, transcriptome co-expression analysis, homology-based prediction, proteomics-based screening, and spatiotemporal expression analysis (Figure 2).

### 2.1. Mining Strategies Based on Metabolic Gene Clusters

Plant metabolic gene clusters (MGCs) generally refer to sets of physically neighboring genes in the genome that participate in the same metabolic pathway. Compared with microorganisms, known MGCs in plants appear to be more limited in distribution and more difficult to identify. Nevertheless, gene clustering has been shown to serve as an important evolutionary and regulatory mechanism in some diterpenoid biosynthetic pathways [25]. Genome mining is one of the major approaches for discovering MGCs. For example, Forman et al. (2022) identified a gene cluster containing five P450 genes in the *Ginkgo biloba* genome [14]. This cluster is tightly linked to the scaffold-forming enzyme gene *GbLPS* and shows root-enriched co-expression. Subsequent functional studies demonstrated that the cluster encodes key oxygenases involved in ginkgolide biosynthesis. In *Salvia miltiorrhiza*, gene family evolutionary analysis enabled the identification of *CYP71D373* and *CYP71D375*, which are located near diterpene synthase genes, and revealed their roles in catalyzing D-ring formation during tanshinone biosynthesis [10].

In addition to linear gene proximity, plants can also coordinate gene regulation through higher-order chromosomal organization. Some metabolic gene clusters may span relatively long linear distances but become spatially associated through three-dimensional chromatin topology, thereby forming coordinated transcriptional states [26]. For example, the gene cluster associated with the *ent*-10-oxodepressin diterpenoid phytoalexin pathway on rice chromosome 7, which includes *OsECBS* and multiple *CYP71Z* genes, exhibits strong co-expression under induced conditions [27]. In recent years, the development of bioinformatic tools such as plantiSMASH 2.0 has improved the efficiency of MGC prediction in plant genomes and has also facilitated the preliminary screening of gene clusters associated with diterpenoid pathways [28].

However, MGCs do not represent a universal pattern of gene organization in plant diterpenoid biosynthesis. Some pathway genes may be dispersed across different chromosomal regions, and genomic proximity does not necessarily indicate functional association. For example, in *Scutellaria barbata*, although some P450 genes are located near diterpene synthase genes, functional validation did not support their involvement in the corresponding diterpenoid pathway [29]. Therefore, candidate gene prediction based solely on genomic location carries inherent risks and generally requires integration with transcriptome co-expression, metabolite accumulation patterns, and functional validation.

### 2.2. Prediction Strategies Based on Co-Expression Analysis

Co-expression analysis is based on the premise that genes involved in the same metabolic pathway often show similar or coordinated expression patterns. By comparing transcriptomic datasets across different tissues, developmental stages, or induced conditions, researchers can identify candidate genes whose expression is highly correlated with known pathway genes or with the accumulation patterns of target metabolites. Compared with MGC mining, co-expression analysis does not depend on genomic location and is therefore better suited for identifying non-clustered metabolic pathway genes.

Inducible expression datasets provide a typical application scenario for co-expression analysis in diterpenoid pathway elucidation. In tanshinone biosynthesis, for example, transcriptome analysis of Ag^+^-induced *S. miltiorrhiza* hairy roots enabled the identification of candidate genes whose expression patterns were highly correlated with the known key enzyme CYP76AH1. This approach led to the characterization of CYP76AH3 and CYP76AK1, which are responsible for ferruginol C-11 hydroxylation and subsequent oxidation reactions [7,9]. Similarly, in *Isodon lophanthoides* var. *graciliflorus*, screening for candidate genes induced by methyl jasmonate (MeJA) and co-expressed with upstream diTPSs led to the identification of several CYP76AH subfamily members involved in the formation of ferruginol derivatives [30]. These examples indicate that, under conditions involving induction, tissue-specific expression, or marked accumulation of target metabolites, co-expression analysis can effectively narrow the candidate gene pool and improve the efficiency of key enzyme screening.

However, co-expression alone does not directly establish enzyme function. For large gene families such as P450s, UDP-dependent glycosyltransferases (UGTs), and BAHD acyltransferases, many genes may respond synchronously under the same treatment condition. These may include enzymes that truly participate in the target pathway, as well as genes associated with stress responses, developmental regulation, or other metabolic processes. Therefore, co-expression analysis usually needs to be combined with metabolite profiling, phylogenetic analysis, and in vivo or in vitro functional validation.

### 2.3. Prediction Strategies Based on Homology

Homology-based prediction based on sequence similarity is a classical approach for mining conserved pathway enzymes. This strategy typically uses functionally characterized enzymes as query sequences and applies algorithms such as BLAST(v2.14.0) and HMMER (v3.3.2) to identify orthologs or paralogs in target species. Candidate genes are then evaluated by integrating expression patterns and functional validation to determine whether they participate in the pathway of interest.

Homology-based prediction is particularly useful for identifying diTPSs. For example, Brückner, Božić, and colleagues used known copalyl diphosphate synthase (CPS) and kaurene synthase-like (KSL) genes as reference sequences and successfully cloned PdiTPS family members involved in the formation of the carnosic acid scaffold from sage and rosemary [31,32]. For downstream modifying enzymes, Zi, Peters, and colleagues used the tanshinone oxidase CYP76AH1 as a template to identify four homologous genes, *CYP76AH4–7*, from the rosemary transcriptome, and confirmed that CYP76AH4 has similar oxidative activity [33].

Nevertheless, homology-based prediction depends strongly on the number and functional coverage of available reference enzymes. For novel scaffolds, unusual modification reactions, or lineage-specific pathways, key enzymes may originate from highly diverged paralogous branches or may show relatively low sequence similarity to known enzymes. In such cases, reliance on sequence similarity alone may miss genuine candidate enzymes or incorrectly assign homologs with similar activities but unrelated pathway roles as target enzymes. Therefore, homology-based prediction is better suited for preliminary screening of conserved reaction types and should not be used as the sole basis for elucidating new and complex diterpenoid pathways.

### 2.4. Functional Screening Based on Proteomics

Unlike transcriptomics, which provides indirect evidence, proteomics can more directly reflect enzyme abundance and biochemical activity. In particular, when combined with chemical probe-based approaches, proteomics can enable targeted capture of substrate-binding proteins and thereby provide evidence that is closer to the functional level for candidate enzyme screening.

In the elucidation of the steviol glycoside pathway, researchers first screened 68 candidate UGTs through global proteomic analysis and then designed an ST-Dayne photoaffinity probe based on the structure of steviol. This probe labeled substrate-binding proteins and ultimately helped identify UGT73E1 as the key enzyme responsible for C-4 glucosylation [34]. This activity-based proteomic strategy, known as activity-based protein profiling (ABPP), provides an effective tool for addressing functional redundancy within large gene families.

Proteomics-based screening also has several limitations. First, target protein abundance, membrane localization, and extraction efficiency can all affect detection. Second, for membrane-associated enzymes such as P450s, protein extraction, maintenance of protein stability, and in vitro activity validation remain technically challenging. Third, chemical probe design depends on prior knowledge of substrate or intermediate structures, whereas key intermediates in many unknown diterpenoid pathways have not yet been defined.

Here, we systematically compare five strategies for discovering genes involved in plant specialized metabolic pathways, as summarized in Table 1. Collectively, these five strategies represent different trade-offs between the efficiency of high-throughput screening and the certainty of functional validation, and their practical application often requires an integrated, multi-strategy approach.

### 2.5. Integrated Strategies Based on Expression Specificity and Evolutionary Associations

The accumulation of PSMs often shows clear tissue specificity, developmental-stage specificity, and inducible responses. Based on the principle of metabolite–transcript co-localization, when a metabolite accumulates strongly in a particular tissue or under a specific condition, candidate enzyme genes that are also highly expressed in the same tissue or condition are more likely to participate in the biosynthesis of that metabolite.

Pateraki et al. focused on root cork cells of *Coleus forskohlii*, which are enriched in forskolin precursors, and used comparative transcriptomics to identify CYP76AH subfamily members and acyltransferases that were specifically highly expressed in this tissue. These genes enabled reconstruction of the forskolin pathway [39]. Similarly, in rosemary, carnosic acid accumulates mainly in young leaves. The high expression of *RoKSL2–RoCPS2* is consistent with the spatial distribution of carnosic acid, and the young leaf-enriched RoCYP76AK7/8 enzymes have also been shown to participate in the biosynthesis of this diterpenoid [40].

However, spatiotemporal expression specificity alone is not sufficient to exclude interference from numerous candidate P450s within the same enzyme family. In recent years, some studies have begun to incorporate molecular evolutionary perspectives in addition to expression-based associations. In elucidating the oridonin pathway, Sun et al. not only examined highly expressed genes in shoot tips, the major biosynthetic site, but also found that the CYP706V subfamily involved in complex oxidative modifications had undergone marked lineage-specific expansion in the genus *Isodon* [41]. Notably, the expansion of the CYP706V subfamily corresponds to the abundant accumulation of kaurane-type diterpenoids in this lineage. This case suggests that the enrichment of specific diterpene scaffolds or scaffold-derived compounds in plant lineages may be associated with the expansion and functional divergence of related modifying enzyme families.

Previous studies have generally suggested that the diversification of PSMs is closely linked to gene duplication, functional divergence or neofunctionalization, and lineage-specific expansion [42,43,44,45]. Therefore, integrating chemotaxonomic and molecular phylogenetic information with conventional co-expression analysis, homology-based prediction, and tissue-specific expression analysis may improve the reliability of prioritization of candidate tailoring enzymes from an evolutionary perspective. This integrated strategy may be particularly useful for complex diterpenoid pathways that are specific to certain genera or families, as it can provide a more targeted set of candidate genes for subsequent functional validation.

## 3. Prioritizing Candidate Tailoring Enzymes Through Lineage-Biased Scaffold Distribution and Gene Family Expansion

The strategies described above have advanced the elucidation of plant-derived diterpenoid biosynthetic pathways from different perspectives. The proposed framework differs from existing workflows mainly in its direction of inference. Metabolic gene cluster mining, co-expression analysis, homology-based prediction, proteomics-based screening, and spatiotemporal expression analysis are primarily gene-centered or enzyme-centered approaches: they first identify candidate genes and then evaluate whether these candidates explain the formation of target metabolites. By contrast, the framework proposed here is metabolite-centered: it first examines whether particular diterpene scaffolds or scaffold-derived compounds are enriched in specific plant lineages, and then evaluates whether relevant tailoring enzyme families show lineage-specific expansion, divergence, or expression patterns, providing evolutionary context for interpreting these chemical distributions. MGC mining facilitates the identification of pathway genes that are physically clustered in the genome; co-expression analysis helps screen candidate genes with coordinated expression patterns; homology-based prediction is useful for tracing enzymes involved in conserved reaction types; and proteomics can provide evidence that is closer to the functional level. However, most of these approaches still begin with “genes or enzymes,” that is, they first search for potential pathway enzymes within candidate gene sets and then validate their functions experimentally. For large modifying enzyme families such as P450s, which often show broad substrate adaptability and rapid functional divergence, sequence similarity, expression correlation, or genomic location alone is insufficient to fully explain the lineage-biased patterns of plant diterpenoid chemical diversity. It is therefore useful to further consider metabolite structures and lineage distributions themselves, and to connect the enrichment of specific diterpene scaffolds with the expansion and divergence of candidate modifying enzyme families. Based on this rationale, this review proposes a data-driven framework that uses the distribution of diterpene core scaffolds as a guide and gene family expansion as an important reference for prioritizing candidate tailoring enzymes.

### 3.1. Data Curation, Scaffold Extraction, Structural Annotation, and Statistical Analysis of Lineage-Associated Diterpenoid Distributions

To investigate the lineage distribution of plant-derived diterpenoids from a structural perspective, we systematically collected and curated structural information, source information, and scaffold characteristics of diterpenoid compounds from publicly available natural product databases and published literature up to June 2025. The initial dataset contained 64,132 diterpenoid-related records. Records were retained only when they met the following criteria: (i) clear plant-derived source information was available; (ii) sufficient structural information was provided to determine the core carbon skeleton; and (iii) the carbon framework met the C20 diterpenoid criterion. Records lacking reliable structural information, organismal source information, plant origin, or a definable core carbon scaffold, as well as obvious non-diterpenoid entries, were excluded.

Data redundancy was removed through a multi-step curation workflow based on compound names (including common synonyms), molecular formulas, structural identifiers (SMILES and InChIKey where available), carbon-number features, and source records. Ambiguous or borderline cases were manually inspected. Exact duplicate database entries were consolidated, whereas records linking the same compound to different plant sources were retained as distinct compound–source records. After curation, 45,177 plant-derived diterpenoid records were retained, representing 589 canonical C20 scaffolds (Supplementary Table S1).

The carbon skeletons of diterpenoids were extracted using RDKit following a previously reported three-step procedure [46]. First, bonds between carbon atoms and heteroatoms were cleaved to separate the original molecular structures into multiple carbon-containing fragments. Second, all bonds within each fragment were converted into single bonds, with stereochemical information removed. Finally, the largest fragment containing exactly 5n carbon atoms (*n* = 4, corresponding to 20 carbons) was selected as the core carbon scaffold of each diterpenoid (Supplementary Table S2). This procedure enables structural comparison based on carbon skeleton topology rather than variations in oxidation state, substitution patterns, or stereochemistry.

To further evaluate structural diversification associated with oxidative modification, the oxidation level of diterpenoid molecules was estimated based on oxygen-containing functional groups and oxidation sites located on the core carbon scaffold. Oxygen-containing sites were classified according to their bonding patterns. Carbon atoms bonded to oxygen through a single bond were classified as hydroxylation sites, carbon atoms double-bonded to oxygen were classified as carbonylation sites, and carboxyl groups containing both C–O and C=O bonds were classified as carboxylation sites. The degree of molecular oxygenation was approximated using the oxygen-to-carbon ratio. All structural calculations were performed using RDKit, and complete oxidation-related results are provided in Supplementary Table S3.

Taxonomic information was standardized based on the original source records of each compound and cross-referenced with accepted plant taxonomy where possible. Species-level information was organized into genus, family, and higher taxonomic categories, enabling comparative analyses of diterpenoid scaffold distributions and structural characteristics among different plant lineages. Dataset integrity was further evaluated through cross-checking against published diterpenoid reviews and manual inspection of randomly selected records.

A reproducible statistical workflow was implemented to quantify lineage-associated scaffold distributions. Undefined and unclassified taxonomic labels were excluded from inferential lineage analyses. For the overall association analysis, lineages represented by at least 50 records and scaffolds represented by at least 20 records were retained. Pearson contingency statistics were calculated; because 86.9–94.8% of expected cell counts were below five, significance was determined by fixed-margin Monte Carlo/permutation sampling (999 replicates for family and genus analyses and 499 replicates for the species-label analysis). Cramér’s V was reported as the association effect size.

For each eligible lineage–scaffold pair, a one-sided hypergeometric test, equivalent to a one-sided Fisher exact test under fixed margins, compared the observed count with the count expected from the complete 45,177-record empirical background. *p* values were adjusted separately at the family, genus, and species-label levels using the Benjamini–Hochberg procedure. Reported enriched pairs required at least five observed records, fold enrichment greater than one, and *q* < 0.05. Extremely small probabilities below double-precision reporting limits are reported as *q* < 1 × 10^−300^. Extremely small q values reflect statistical overrepresentation within the curated record-level dataset and should not be interpreted as correspondingly strong biological evidence.

Because database and literature records are non-independent and are influenced by research focus, species availability, analytical accessibility, database coverage, and publication bias, the curated dataset does not represent a uniform or random survey of plant diterpenoid diversity. Record-level permutation and enrichment analyses were therefore complemented by a binary named-species occurrence analysis, in which each species–scaffold combination was counted once. Spearman correlations between lineage record count and scaffold richness were used to assess potential sampling effects. These analyses quantify overrepresentation relative to the curated empirical background but do not estimate absolute abundance in nature or establish enzyme function or evolutionary causality. The resulting patterns should therefore be treated as hypotheses requiring validation through broader taxonomic sampling, phylogenetically informed comparative analyses, genomics, expression profiling, biochemical characterization, and genetic experiments.

### 3.2. Lineage Preferences and Oxidative Modification Features of Plant-Derived Diterpene Scaffolds

**Figure 3:**
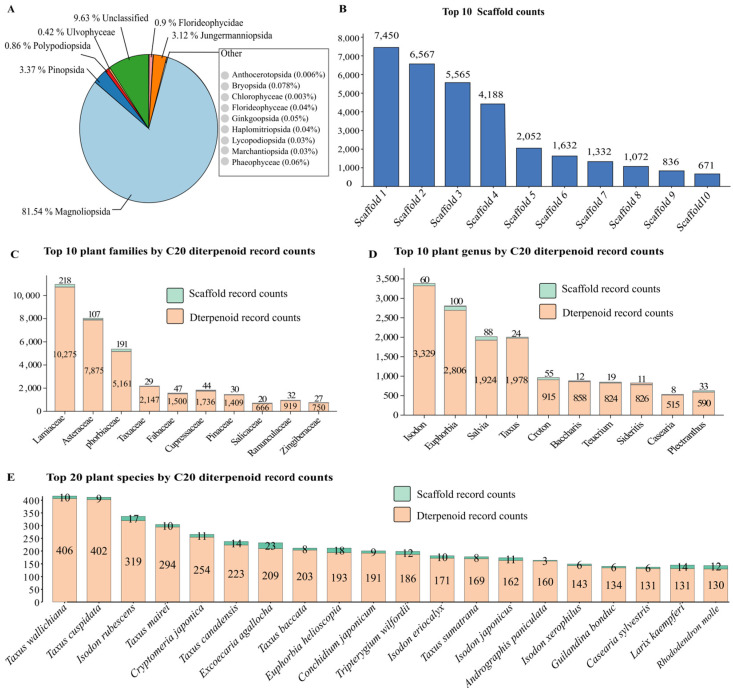
Counts of curated diterpenoid compound–source records and canonical scaffold diversity across taxonomic groups. (**A**) Distribution of curated diterpenoid records among major source groups. (**B**) The ten most frequently reported canonical C20 scaffolds; the corresponding structures are provided in Supplementary Table S2. (**C**–**E**) Curated diterpenoid compound–source record counts and canonical scaffold counts in the top 10 families, top 10 genera, and top 20 species ranked by diterpenoid record count, respectively; detailed data are provided in Supplementary Table S4. The reported record counts represent minimum estimates based on currently available database and literature records and should not be interpreted as absolute abundances in nature.

Based on the curated dataset, source statistics indicate that green plants (Viridiplantae) represent a major reported source of natural diterpenoid diversity. In the original diterpenoid records, green plants accounted for a relatively high proportion of the dataset compared with mammals, fungi, bacteria, and other groups (Figure 3A). Although this proportion may be influenced by research attention in natural product chemistry and the scope of database coverage, it nevertheless suggests that plants represent an important source of diterpenoid chemical diversity. Among the 45,177 plant-derived compound–source records, 589 core skeletons conforming to the C20 rule were identified. Linear, bicyclic, tricyclic, tetracyclic, and macrocyclic scaffolds constituted the major scaffold types (Figure 3B) and showed certain lineage-biased patterns across plant groups. For example, bicyclic scaffolds were relatively abundant in Asteraceae and Taxaceae, tetracyclic kaurane-type scaffolds were prominent in the genus *Isodon* within Lamiaceae, and macrocyclic scaffolds were mainly found in Euphorbiaceae and *Taxus*-related groups. Further comparison of compound numbers and scaffold-type numbers showed that, in some plant groups, a large number of diterpenoid compounds are often derived from a relatively limited number of scaffold types. For example, in Lamiaceae, the ratio between compound number and scaffold-type number was relatively high, with an average of approximately 50 compounds corresponding to one scaffold type (Figure 3C–E). This pattern suggests that the diversification of plant diterpenoids does not depend solely on the continual generation of new carbon scaffolds, but may rely more strongly on the sustained modification and derivatization of existing core scaffolds. Therefore, “diterpene scaffold conservation” does not imply a lack of scaffold variation. Rather, it indicates that, within certain plant lineages, a limited set of core scaffolds can give rise to numerous derivatives through post-scaffold modification reactions.

Quantitative analysis supported a non-random association between taxonomic lineage and common scaffold distribution. At the family level, 40,005 records from 59 eligible families and 98 common scaffolds yielded Cramér’s V = 0.465 (permutation *p* ≤ 0.001). At the genus level, 32,795 records from 152 genera and 100 scaffolds yielded Cramér’s V = 0.484 (*p* ≤ 0.001). The species-label analysis included 15,256 records, 151 labels, and 94 scaffolds and yielded Cramér’s V = 0.477 (*p* ≤ 0.002). Because 86.9–94.8% of expected cells were below five, permutation *p* values rather than asymptotic chi-square *p* values were used for inference (Supplementary Table S8).

Pairwise enrichment tests identified lineage–scaffold combinations contributing to the overall association. In *Isodon*, kaurane-type scaffold 2 occurred 2351 times compared with 483.9 expected under the complete empirical background (4.86-fold enrichment; odds ratio = 21.46; BH *q* < 1 × 10^−300^). Other illustrative associations included abietane-type scaffold 4 in *Salvia* (999 observed versus 178.4 expected; 5.60-fold; odds ratio = 13.57; *q* < 1 × 10^−300^), scaffold 9 in *Euphorbia* (784 versus 51.9; 15.10-fold; odds ratio = 315.55; *q* < 1 × 10^−300^), and scaffold 20 in *Taxus* (342 versus 17.3; 19.83-fold; odds ratio = 173.46; *q* < 1 × 10^−300^) (Supplementary Tables S9–S11).

The named-species occurrence analysis provided a conservative sensitivity assessment. Scaffold 2 was recorded in 57 of 60 named *Isodon* species, compared with 11.5 species expected from the empirical species background (4.97-fold enrichment; odds ratio = 88.11; *q* = 1.38 × 10^−35^). Scaffold 4 was recorded in 102 of 155 named *Salvia* species (4.57-fold; odds ratio = 14.18; *q* = 2.52 × 10^−48^). Representative *Euphorbia* and *Taxus* associations were also retained (Supplementary Table S12).

The statistical results require cautious interpretation. Observed scaffold richness was strongly correlated with record count at the family (Spearman’s *ρ* = 0.850, *p* = 7.32 × 10^−71^), genus (*ρ* = 0.750, *p* = 1.72 × 10^−219^), and species-label levels (*ρ* = 0.615, *p* < 1 × 10^−300^). Thus, the reported associations are database- and literature-supported overrepresentation patterns. They provide quantitative candidate clues for enzyme family prioritization but do not establish natural prevalence, enzyme function, or an evolutionary causal relationship.

### 3.3. An Evidence-Guided Framework for Prioritizing Candidate Tailoring Enzymes by Integrating Scaffold-Derived Chemical Diversity and Gene Family Evolution

Following the descriptive criteria defined in Section 3.2, lineage-biased distributions of diterpene scaffolds and their derivatives can be used to identify potential chemical diversity hotspot lineages. Diterpenoids have long been used as chemotaxonomic markers within Lamiaceae [47]. Structurally diverse kaurane-type diterpenoids are particularly well represented in *Isodon* [48,49]. Although kaurane-type scaffolds are widely distributed throughout the plant kingdom because of their roles in gibberellin biosynthesis, structurally diversified specialized metabolites derived from these scaffolds are particularly well represented in the genus *Isodon*, including *I. rubescens*. Here, “potential chemical diversity hotspot” refers to a lineage showing statistically supported overrepresentation relative to the curated empirical background. It refers to a lineage in which the reported diversity or relative representation of derivatives derived from a particular scaffold is comparatively high. This pattern should not, however, be interpreted as evidence of natural prevalence or evolutionary causality.

**Figure 4:**
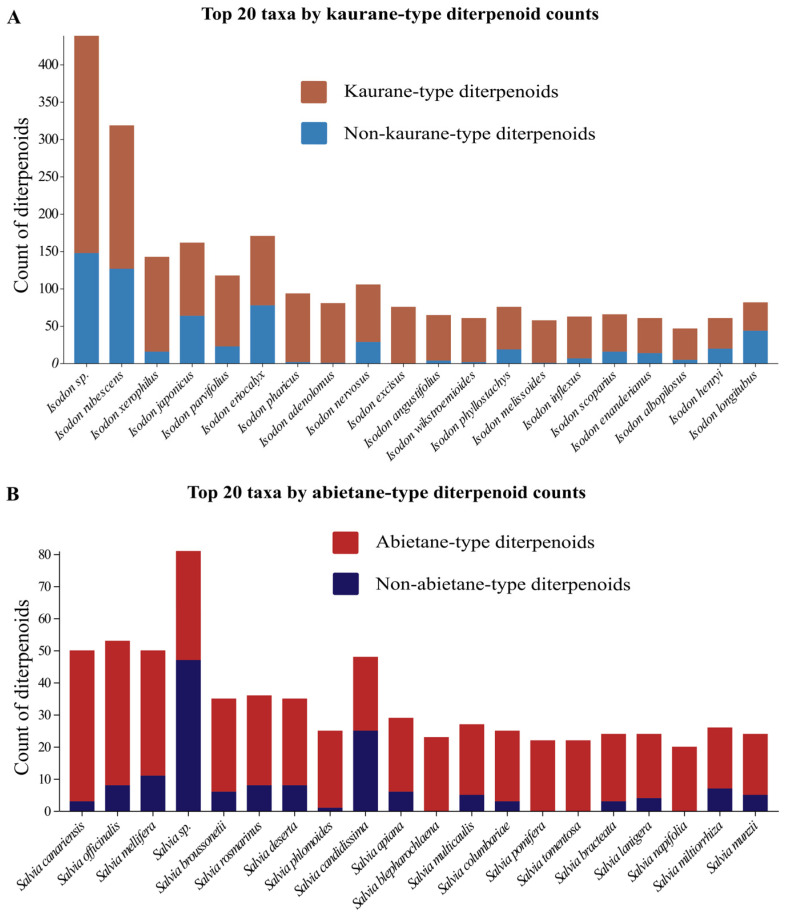
Curated diterpenoid compound–source record counts for the top 20 *Isodon* taxa and the top 20 *Salvia* taxa. (**A**) Kaurane-type and non-kaurane-type diterpenoid record counts for the 20 *Isodon* taxa ranked by kaurane-type diterpenoid record count; detailed data are provided in Supplementary Tables S5 and S6. (**B**) Abietane-type and non-abietane-type diterpenoid record counts for the 20 *Salvia* taxa ranked by abietane-type diterpenoid record count; detailed data are provided in Supplementary Table S7. The stacked bars represent curated compound–source record counts based on currently available database and literature records and should not be interpreted as absolute metabolite abundances in planta.

Representative diterpenoid pathways provide different levels of evidence linking chemical diversification with tailoring enzyme evolution. In *Isodon rubescens*, one of the species particularly rich in kaurane diterpenoids (Figure 4A), Sun et al. identified lineage-specific expansion and tandem duplication of the CYP706V subfamily. Functional studies further demonstrated that these expanded CYP706V members catalyze diverse site-specific oxidations of *ent*-kaurene and its derivatives, representing an example in which gene family evolution and kaurane diterpenoid diversification are supported within the same biosynthetic context [41]. In *Salvia*, abietane-type diterpenoids are relatively well represented in several species in the curated dataset (Figure 4B). Functional divergence within the CYP76AK subfamily, including the retention, modification, and loss of ancestral catalytic activities, has been proposed to contribute to lineage-associated variation in abietane-type diterpenoid profiles [50]. Together with the *Isodon* example, the *Salvia* system supports the biological plausibility of integrating scaffold distributions with gene family evolution for candidate enzyme prioritization. These examples should nevertheless be regarded as evidence-guided case studies rather than universal evolutionary rules.

Collectively, the *Isodon* and *Salvia* studies validate different components of the proposed framework: the *Isodon* example connects lineage-associated gene family evolution with experimentally characterized catalytic functions; the *Salvia* example demonstrates how functional retention, modification, and loss within an enzyme subfamily can contribute to clade-associated metabolic variation. Together, these findings support the biological plausibility of integrating metabolite distributions with enzyme family evolution, but they do not demonstrate that all diterpenoid-rich lineages follow a common expansion–neofunctionalization–chemical enrichment process.

**Figure 5:**
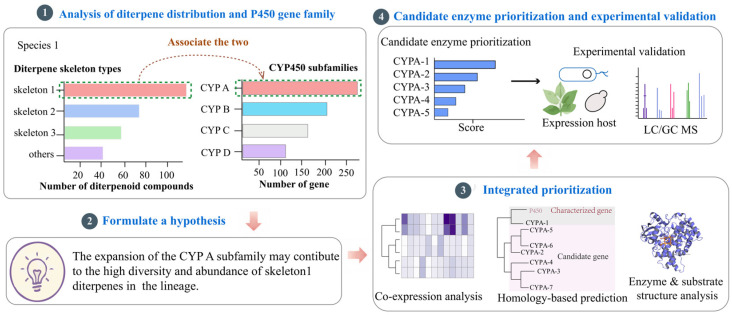
Evidence-guided workflow for prioritizing candidate tailoring enzymes in diterpenoid biosynthesis by integrating lineage-biased scaffold distributions with gene family evolution.

On the basis of these precedents and the descriptive chemical distribution patterns identified in the curated dataset, we propose the following testable hypothesis: in some plant lineages characterized by comparatively high scaffold-derived chemical diversity, downstream tailoring-enzyme families may have undergone lineage-specific expansion, retention, or functional divergence, thereby contributing to their capacity to generate diverse derivatives of particular diterpene scaffolds. This hypothesis does not imply that scaffold enrichment necessarily causes enzyme family expansion, that expansion invariably results in neofunctionalization, or that gene family size alone predicts metabolic diversity. Based on this hypothesis, a metabolite-centered workflow for candidate enzyme prioritization can be proposed (Figure 5).

The central feature of this strategy is therefore not the direct prediction of enzyme function from metabolite distribution. Rather, lineage-biased scaffold-derived diversity provides an additional layer of evidence that can be integrated with comparative gene family evolution and conventional gene-centered analyses to narrow the candidate pool. Enzyme function must ultimately be established through biochemical and/or genetic validation, whereas broader evolutionary relationships require comparative genomic and phylogenetic evidence, appropriately controlled quantitative analyses, and consideration of unequal sampling and research effort.

## 4. Recent Advances in Elucidating Plant-Derived Diterpenoid Biosynthetic Pathways

This section summarizes recent progress in the elucidation of biosynthetic pathways for acyclic, bicyclic, tricyclic, tetracyclic, taxane-type, and macrocyclic diterpenoids based on the ring-system characteristics of their core scaffolds [51,52,53]. Although the elucidation of plant diterpenoid biosynthesis has long been centered on a limited number of well-studied medicinal pathways, such as tanshinones, forskolin, oridonin, ginkgolides, and paclitaxel [3,9,14,39,41,54], recent studies have increasingly expanded pathway discovery to phylogenetically diverse and less-studied plant lineages, including furanoditerpenoids from *Panicum virgatum* [55], marrubiin from *Marrubium vulgare* [56], clerodane diterpenoids from *Scutellaria* [29], salvinorin A from *Salvia divinorum* [57], macrocyclic diterpenoids from Euphorbiaceae [58], and diterpenoid alkaloids from *Aconitum* [59]. These examples demonstrate that diterpenoid pathway discovery is no longer limited to a few canonical medicinal compounds. Instead, comparative genomics, transcriptomics, metabolomics, co-expression analysis, and phylogenetic analysis are increasingly revealing pathway enzymes from non-model or less-studied lineages. Rather than listing compounds individually, this section focuses on the common biosynthetic logic of different scaffold classes, the major types of modifying enzymes involved, and the implications of these pathways for the proposed candidate enzyme prioritization framework based on scaffold distribution and gene family expansion.

### 4.1. Biosynthesis of Acyclic Diterpenoids

**Figure 6:**
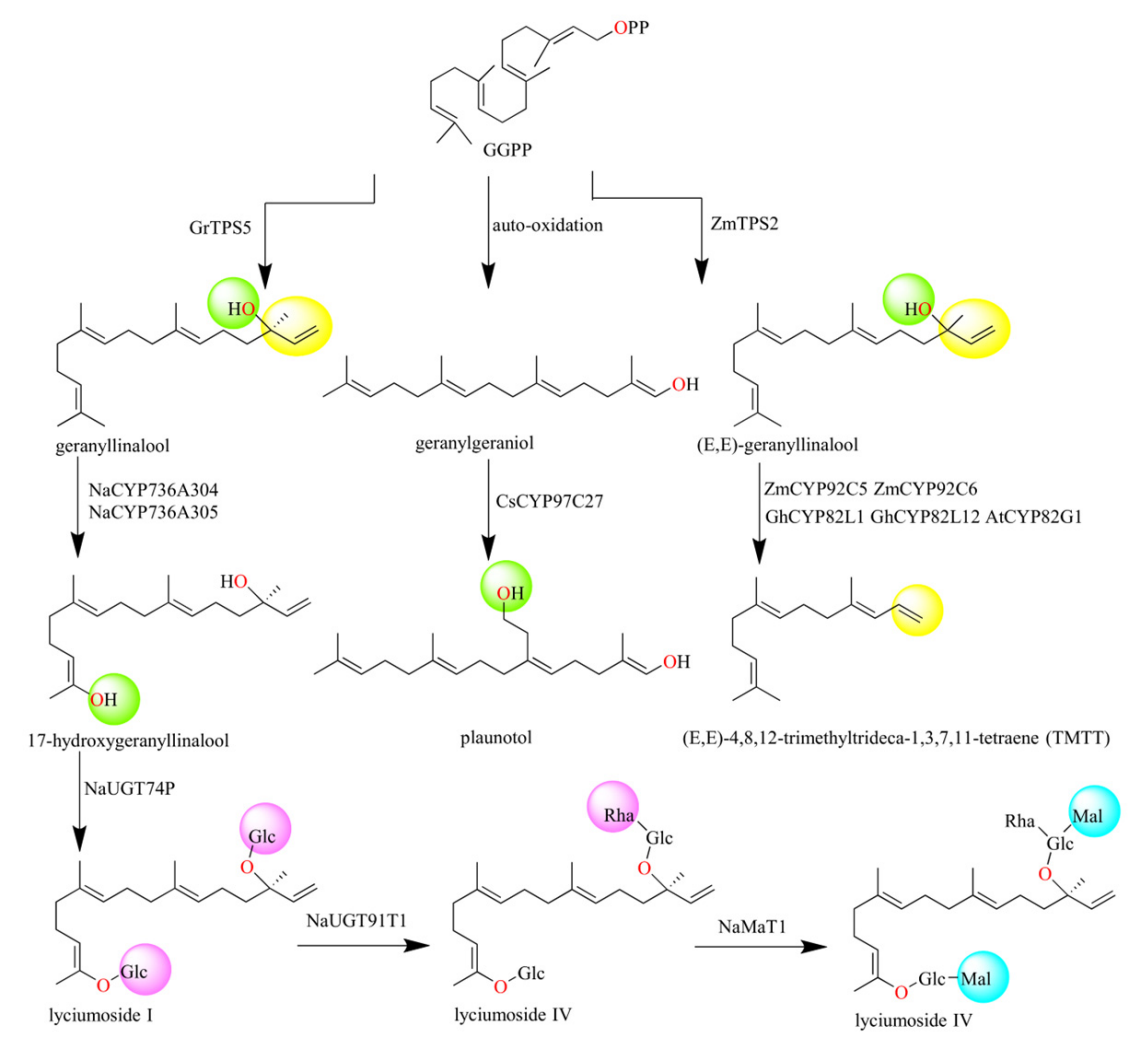
Characterized diTPSs and P450s in the biosynthesis of acyclic diterpenoids. In the structures, Glc, Rha, and Mal denote glucosyl, rhamnosyl, and malonyl groups, respectively. Comprehensive data are available in Supplementary Tables S13–S16.

Acyclic diterpenoids retain linear carbon-chain structures, and their chemical diversity is mainly generated through terminal oxidative modifications. Multiple plant-derived diTPSs have been shown to convert GGPP into geranyllinalool (GL) (Figure 6).

Representative enzymes include GrTPS5 from *Grindelia robusta* [60], NaGLS from *N. attenuate* [61], AtGES from *Arabidopsis thaliana* [62], ZmTPS2 from *Zea mays* [63], and TwGES from *Tripterygium wilfordii* [64]. Downstream modifications in acyclic diterpenoid pathways are typically coordinated by P450s and glycosyltransferases, which together regulate product activity and toxicity. In tobacco, for example, GL undergoes hydroxylation by CYP736A family members, followed by glycosylation catalyzed by the UGT74P family, ultimately producing the defensive metabolites 17-HGL-DTGs [20]. In *Croton stellatopilosus*, *Z*. *mays*, and *Gossypium hirsutum*, members of the CYP97C [65], CYP92C [63], and CYP82G [66] families have been identified as participating in the biosynthesis of plaunotol and the volatile signaling molecule 4,8,12-trimethyltrideca-1,3,7,11-tetraene (TMTT), a C16-homoterpene. Overall, modifying enzymes involved in acyclic diterpenoid pathways are frequently associated with plant defense and volatile signal production, providing useful clues for screening candidate P450s and UGTs from tissue-specific or inducible expression datasets.

### 4.2. Biosynthesis of Bicyclic Diterpenoids

**Figure 7:**
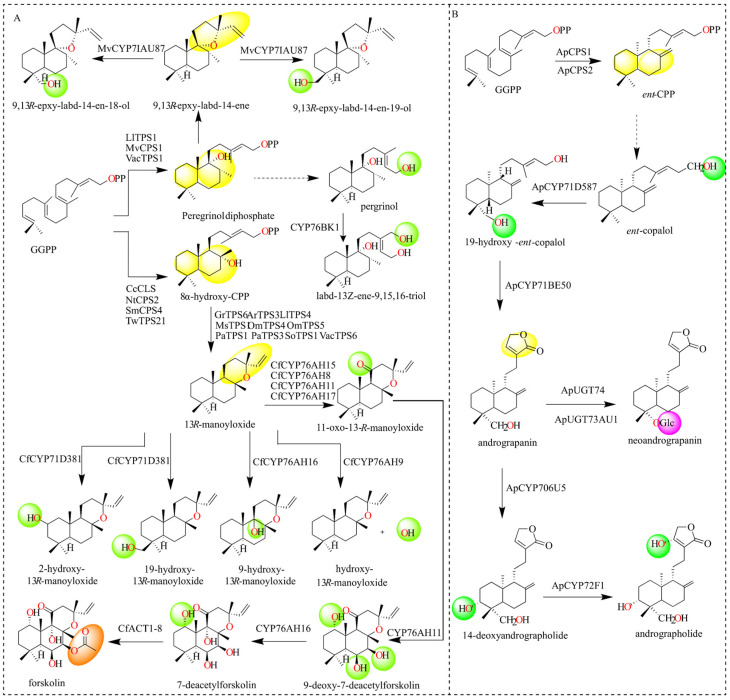
(**A**) Characterized diTPSs and P450s in the biosynthesis of bicyclic diterpenoids. (**B**) The biosynthetic pathway for andrographolide and the enzymes whose functions have been characterized in the pathway. The “Glc” represents the glucose group. Comprehensive data are available in Supplementary Tables S13–S16.

The increased stereochemical complexity of bicyclic diterpenoids provides additional sites for downstream modification. Forskolin and andrographolide are among the best-characterized examples of this scaffold class (Figure 7A).

Forskolin accumulates specifically in root cork cells of *C. forskohlii*. Its biosynthesis begins with scaffold formation by CfTPS2 and CfTPS3 and is followed by coordinated modifications catalyzed by CYP76AH subfamily enzymes and acyltransferases, ultimately yielding the final product [11]. In contrast, andrographolide biosynthesis is initiated by ApCPS1/2-catalyzed conversion of GGPP into *ent*-copalyl diphosphate, after which downstream oxidative networks and UDP-dependent glycosyltransferases contribute to structural maturation (Figure 7B) [12,13,14]. In addition, several P450 enzymes identified in *Vitex agnus-castus*, *Panicum virgatum*, and *Marrubium vulgare* are available in Supplementary Table S14.

### 4.3. Biosynthesis of Tricyclic Diterpenoids

**Figure 8:**
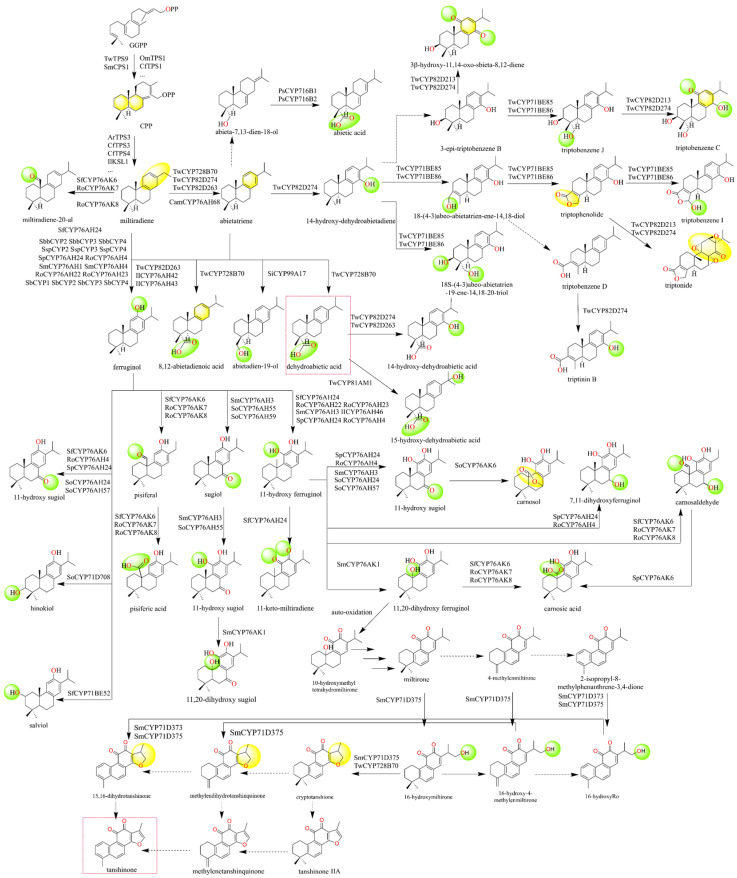
Biosynthesis of abietane-type tricyclic diterpenoids. The red box highlights the target end products of tanshinone and carnosic acid biosynthesis. Solid arrows indicate biochemically characterized steps; dashed arrows indicate uncharacterized steps; and multiple arrows indicate multistep reactions. Comprehensive data are available in Supplementary Tables S13–S16.

Tricyclic diterpenoids mainly include abietane- and pimarane-type scaffolds. Representative products include tanshinones, carnosic acid, triptolide, triptonide, ginkgolides, and rice diterpenoid phytoalexins. These scaffolds are generally constructed by CPS/KSL-type diterpene synthases and then undergo complex modifications, including sequential oxidation, aromatization, lactonization, ring rearrangement, or C–C bond cleavage. They therefore provide important systems for studying functional divergence among P450 enzymes. Among abietane-type diterpenoids, the biosynthetic pathways of tanshinones, carnosic acid, triptolide, and triptonide represent well-characterized examples (Figure 8).

**Figure 9:**
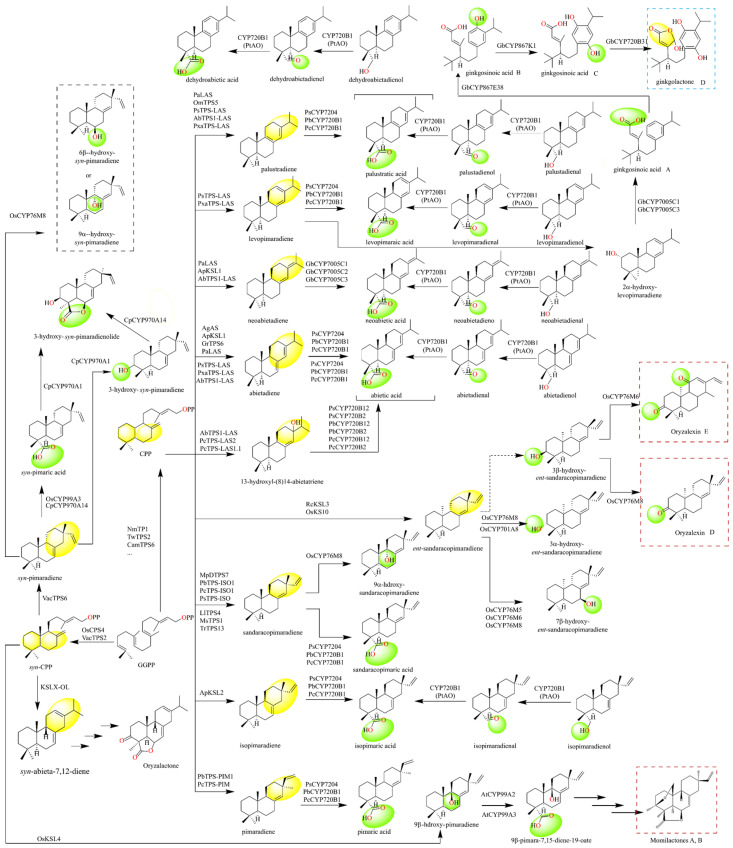
Summary of the biosynthesis of characterized pimarane-type tricyclic diterpenoids. Red boxes indicate end products accessible through the currently reconstructed diterpenoid phytoalexins pathway. Blue boxes indicate end products accessible through the currently reconstructed ginkgolide pathway. Comprehensive data are available in Supplementary Tables S13–S16.

In *S. miltiorrhiza*, SmCPS1 and SmKSL1 construct the tanshinone diene scaffold, after which enzymes from the CYP76AH [6,7,8], CYP76AK [9], and CYP71D [10] subfamilies form a complex oxidative network that mediates a series of reactions from aromatization to D-ring lactonization. In the carnosic acid pathway, members of the CYP76AH and CYP76AK subfamilies show clear substrate promiscuity and can convert ferruginol into different products, such as pisiferic acid. Although triptolide and triptonide are also abietane-related diterpenoids, their downstream modification routes have diverged and involve enzymes such as CYP728B70 [67], CYP71BE [68], and CYP82D [69]. Progress in the study of pimarane-type compounds has mainly focused on ginkgolides and phytoalexins (Figure 9).

Forman et al. elucidated key early steps in the biosynthesis of ginkgolides [14]. This pathway is initiated by GbLPS and proceeds through a complete route cooperatively catalyzed by five co-expressed P450 enzymes, including CYP7005C, CYP867E/K, and CYP720B. The pathway involves unusual C–C bond cleavage and sequential hydroxylation, ultimately producing ginkgolides C and D. The formation of rice diterpenoid phytoalexins depends on sequential oxidation catalyzed by lineage-specific P450 systems, including CYP76M [70,71], CYP99A [72], and CYP71Z [73] enzymes. Recent functional characterization of KSLX-OL and the CYP71Z family has further refined the metabolic map of defensive diterpenoids in rice [74,75]. Overall, these metabolic networks are also consistent with a biosynthetic logic in which specific P450 subfamilies modify particular scaffolds to expand diterpenoid diversity.

### 4.4. Biosynthesis of Tetracyclic Diterpenoids

**Figure 10:**
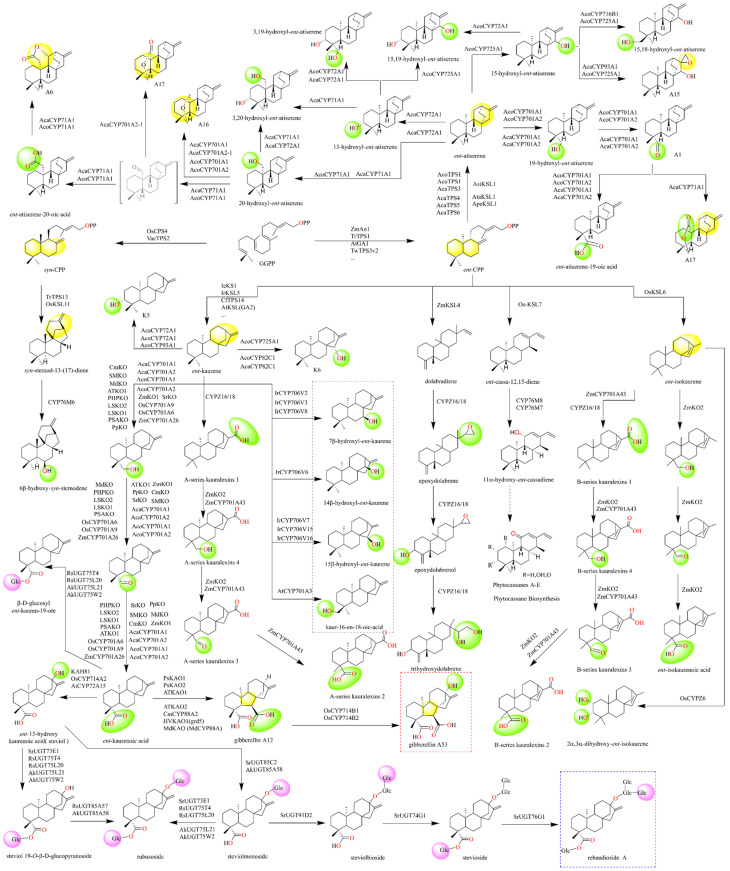
Summary of biosynthesis of characterized tetracyclic diterpenoids. The red box indicates end products accessible through the currently reconstructed gibberellin pathway. Blue boxes indicate end products accessible through the currently reconstructed stevioside pathway. Gray boxes indicate currently synthesizable intermediate products in the biosynthesis pathway of oridonin. Comprehensive data are available in Supplementary Tables S13–S16.

Representative tetracyclic diterpene scaffolds include the *ent*-kaurane- and *ent*-atiserane-type scaffolds (Figure 10).

The *ent*-kaurane scaffold serves as a precursor in gibberellin biosynthesis and reflects an evolutionary branch point from primary metabolism toward specialized metabolism [76]. In *Stevia rebaudiana*, intermediates from the gibberellin pathway are recruited and modified through hydroxylation by KA13H and a series of glycosylation reactions catalyzed by glycosyltransferases such as SrUGT85C2 and SrUGT74G1, leading to the formation of high-intensity sweet steviol glycosides [16].

An alternative glycosylation route requiring two additional steps has also been identified in *Rubus suavissimus* and *Angelica keiskei* [77]. In addition, in *I. rubescens*, *ent*-kaurene is subjected to site-specific oxidation by the lineage-expanded CYP706V subfamily to produce oridonin, which has strong anti-inflammatory activity [41]. This pathway shows a clear evolutionary divergence from the gibberellin biosynthetic route, in which post-scaffold modification is mainly mediated by CYP701. In *I. rubescens*, the kaurane scaffold is generated through the coordinated activity of IrCPS4/5 and IrKSL5, whereas the subsequent key oxidation steps are primarily mediated by the CYP706V P450 subfamily that has specifically expanded in this species [41,54].

For *ent*-atiserene-type diterpenoids, Luo et al. (2025) made substantial progress in elucidating diterpenoid biosynthesis in plants of the genus *Aconitum* [78]. They identified nine diTPSs and 14 P450 enzymes from *Aconitum carmichaelii* and *Aconitum coreanum*. Using a combinatorial biosynthesis strategy, they successfully constructed tripterifordin, guan-fu diterpenoid A, and 14 new atiserenoid diterpenes in a yeast chassis. This study demonstrates the combinatorial potential of P450 enzymes in multi-site scaffold oxidation.

### 4.5. Biosynthesis of Taxane-Type Diterpenoids

**Figure 11:**
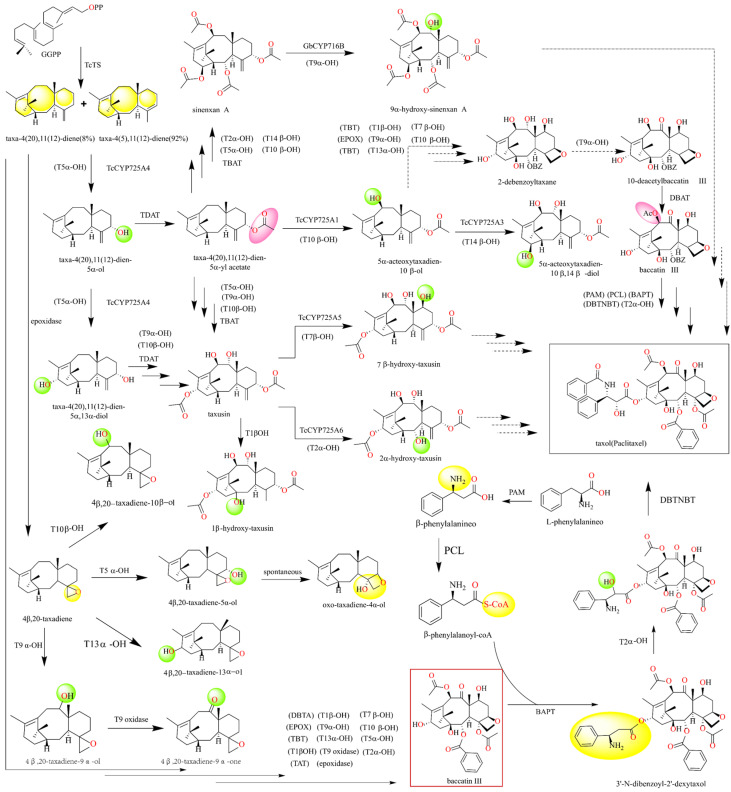
Biosynthesis of paclitaxel. The red box shows baccatin III, an important precursor in the biosynthesis of paclitaxel. The black box shows paclitaxel, the end product. TXS: taxadiene synthase; T5*α*OH: taxane 5*α*-hydroxylase; TAT: taxadiene-5*α*-ol-O-acetyltransferase; T10*β*OH: taxane 10*β*-hydroxylase; T13*α*OH: taxane 13α-hydroxylase; T2*α*OH: taxane 2*α*-hydroxylase; T9*α*OH: taxane 9*α*-hydroxylase; T7*β*OH: taxane 7*β*-hydroxylase; T1*β*OH: taxane 1*β*-hydroxylase; TBT: taxane-2*α*-O-benzoyltransferase; DBAT: 10-deacetyl baccatin III-10-O-acetyltransferase; BAPT: baccatin III-3-amino,13-phenylpropanoyltransferase; DBTNBT: 3′-N-debenzoyl-2′-deoxytaxol-N-benzoyltransferase; PAM: phenylalanine aminomutase; PCL: β-phenylalanine coenzyme A ligase. Comprehensive data are available in Supplementary Tables S13–S16.

Taxane-type diterpenoids are best exemplified by paclitaxel, whose characteristic 6/8/6 tricyclic scaffold and highly oxygenated structure have made its biosynthesis a longstanding challenge in natural-product research [79]. The pathway begins with taxadiene synthase (TXS), which converts geranylgeranyl diphosphate into taxa-4(5),11(12)-diene [80]. Following decades of investigation, several recent landmark studies have resolved key steps that were previously unclear (Figure 11). In particular, CYP725A4 was identified as the enzyme responsible for the formation of the characteristic oxetane ring, leading to a revision of earlier models concerning the timing of oxetane formation during paclitaxel biosynthesis [79]. Subsequently, Zhang et al. discovered the previously unidentified C1 hydroxylase (TOT) and C9 oxidase through heterologous pathway reconstruction, which reduced the core enzyme set for baccatin III biosynthesis to nine [3,81]. Elucidation of the taxane pathway illustrates that complete reconstruction of complex diterpenoid pathways often depends on the coordinated integration of multiple P450s, acyltransferases, and heterologous expression systems. Because taxane diterpenoids are strongly enriched within the genus *Taxus*, these pathways also support the strategy of tracing candidate modifying enzyme families from lineages showing scaffold-specific enrichment.

### 4.6. Biosynthesis of Macrocyclic Diterpenoids

Macrocyclic diterpenoids, which contain carbon rings of seven or more members, are mainly distributed in Euphorbiaceae species [82,83]. Casbene serves as a central precursor for this class of compounds. Kirby et al. (2010) were the first to identify casbene synthases (CBSs) from Euphorbiaceae species, including *Euphorbia esula*, *Euphorbia resinifera*, *Sapium sebiferum*, and *Ricinus communis* [15]. Subsequent genome-mining studies revealed that macrocyclic diterpenoid biosynthesis is often encoded by gene clusters, in which CBS, CYP726A family enzymes, and alcohol dehydrogenases (ADHs) collectively participate in the formation of casbene-derived metabolites.

**Figure 12:**
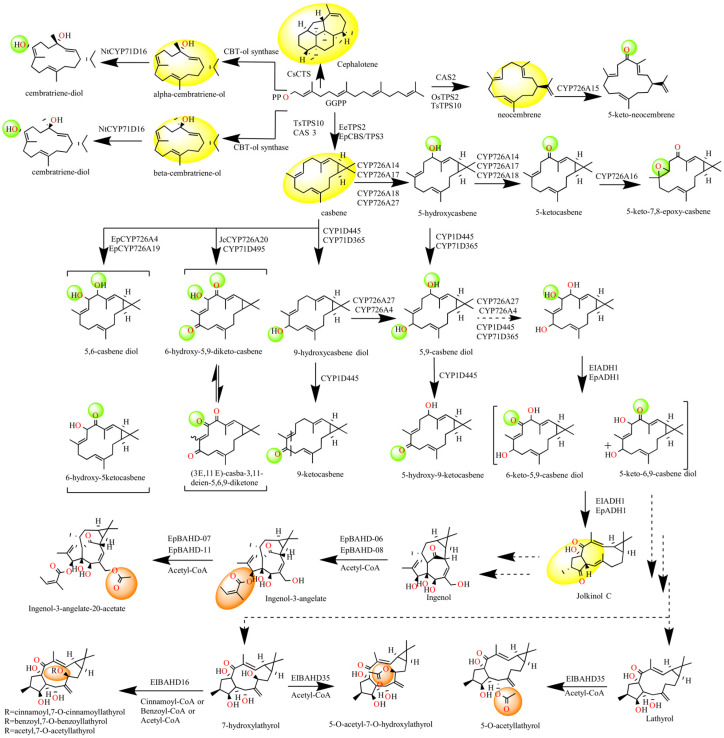
Characterized diTPSs and P450s, together with their substrates and products, in macrocyclic diterpenoid biosynthesis. Comprehensive data are available in Supplementary Tables S13–S16.

In *Euphorbia lathyris* and *Euphorbia peplus*, pathway elucidation has focused primarily on post-scaffold modifications (Figure 12). CYP71D445 and CYP726A27, together with ElADH1, catalyze rearrangement and cyclization reactions leading to formation of jolkinol C, a key intermediate of ingenane-type diterpenoids [58,84]. More recent advances have focused on the identification of BAHD acyltransferases. Schotte et al. (2025) and Zhao et al. (2025) independently characterized BAHD enzymes involved in the scaffold-specific acylation of ingenol- and lathyrane-type diterpenoids, respectively [85,86]. These studies highlight the role of acylation in expanding macrocyclic diterpenoid structural diversity. Collectively, the lineage-biased distribution of these compounds within the Euphorbiaceae appears to be intimately linked to the functional divergence of the associated P450, ADH, and BAHD enzyme families. In addition, the availability of genomic resources for multiple Euphorbiaceae species makes this family another suitable system for evaluating the scaffold enrichment–gene family expansion framework proposed in this review for predicting pathway enzymes.

## 5. Conclusions and Future Perspectives

This review integrates the structural diversity, lineage-associated distribution, scaffold types, and oxidative modification features of plant-derived diterpenoids to address a central challenge in diterpenoid biosynthesis: identifying the enzymes responsible for scaffold formation and downstream modification from large candidate enzyme families. From 64,132 diterpenoid-related structures and source records, 45,177 curated plant-derived diterpenoid records were retained, representing 589 canonical C20 scaffolds. Their distribution was highly uneven across plant lineages, with heavily studied taxa contributing a substantial proportion of the available records.

Quantitative analyses demonstrated significant associations between taxonomic lineage and common scaffold distribution at the family, genus, and species-label levels, with Cramér’s V values of 0.465, 0.484, and 0.477, respectively. Representative enriched lineage–scaffold relationships included kaurane-type scaffold 2 in *Isodon*, abietane-type scaffold 4 in *Salvia*, scaffold 9 in *Euphorbia*, and scaffold 20 in *Taxus*. These patterns were retained in the more conservative named-species occurrence analysis, supporting lineage-associated organization of currently documented diterpenoid diversity. However, scaffold richness was also strongly correlated with record number, indicating that the observed patterns reflect both biological specialization and variation in sampling intensity, database coverage, and research effort. They should therefore be interpreted as database- and literature-supported overrepresentation patterns rather than estimates of natural abundance or evidence of evolutionary causality.

Within these limitations, integrating scaffold distribution, lineage enrichment, oxidative modification features, and enzyme family evolution provides an evidence-guided framework for prioritizing candidate enzymes, including diTPSs, P450s, acyltransferases, and glycosyltransferases. Broadly enriched scaffolds may direct attention toward conserved or expanded enzyme clades, whereas lineage-restricted oxidative derivatives may highlight recently diversified tailoring enzymes. This framework is intended to complement, rather than replace, gene-cluster mining, co-expression analysis, homology-based prediction, structural modeling, and experimental validation. Accordingly, scaffold enrichment and enzyme family expansion should be treated as a prioritization hypothesis until supported by genomic, transcriptomic, biochemical, and genetic evidence.

Future progress will depend on translating lineage-level hypotheses into experimentally verified enzyme functions and expanding catalytic diversity beyond that sampled by natural evolution. Three directions are particularly important: improving substrate–enzyme mapping, applying generative approaches to enzyme design, and integrating computational discovery, enzyme engineering, and experimental validation into an iterative closed-loop workflow.

### 5.1. From Finding Similar Sequences to Predicting Substrate–Enzyme Compatibility

For downstream modifying enzymes such as P450s, the key issue is not merely whether a candidate gene belongs to a known enzyme family, but whether its active site can accommodate a specific diterpenoid scaffold, stabilize a particular reaction conformation, and catalyze selective reactions at specific carbon atoms. Computational resources and heme-guided studies—including PCPD [87], PlantP450Dock [88], and the identification of *ent*-kaurene C-14-hydroxylating P450s [89], have demonstrated that heme incorporation, substrate docking, target carbon–heme distances, MD stability, and MM/PBSA decomposition can significantly enhance the interpretability of candidate P450 screening. In addition, general enzyme–substrate models such as ESP [90], EZSpecificity [91], and EnzymeCAGE [92] suggest that future functional prediction of diterpenoid P450s could further integrate protein language models, substrate 3D conformations, active site geometry, and evolutionary information. This approach advances the predictive goal from “whether an enzyme may act on a class of diterpenoids” to “whether an enzyme can catalyze oxidation at specific carbon atoms of a specific diterpenoid scaffold.”

To achieve more effective substrate–enzyme mapping, it is also necessary to develop diterpenoid representation systems that connect chemical structures with biosynthetic logic. Traditional SMILES, molecular fingerprints, or general structural similarity metrics are suitable for database searches, but they struggle to encode diterpenoid skeleton rearrangements, conserved carbon atom correspondences, oxidation site positions, and possible reaction sequences. Previous studies on triterpenoids have attempted to establish a unified representation of core skeletons and modification sites by standardizing atom and bond position encoding, providing a computational reference framework for terpenoid natural products [93]. Extending this concept to diterpenoids could establish a standardized diterpenoid scaffold coordinate system to encode conserved carbon atoms, ring junctions, migrating bonds, oxidation sites, and glycosylation or acylation positions. If this system is further integrated with known diterpene synthase and modifying enzyme reaction rules, it may allow inference of potential biosynthetic pathways from metabolite structures and provide more mechanistically interpretable inputs for candidate enzyme ranking.

### 5.2. From Predicting Enzymes to Designing or Engineering Enzymes

Substrate–enzyme mapping can reveal structural determinants of regioselectivity and substrate compatibility, providing useful constraints for P450 engineering and active-site design [94]. Existing cases of diterpenoid P450s provide preliminary support for this design logic. For example, heme-guided screening identified bacterial P450s capable of C-14 hydroxylation of *ent*-kaurane diterpenoids by evaluating the distance between the target carbon and the heme iron [89]. Molecular-dynamics simulations further showed that productive catalysis requires a stable, catalytically favorable substrate orientation rather than a plausible static docking pose alone [89]. Similarly, mutations in CYP76AH1 altered substrate binding and expanded its oxidation profile from mainly C12 oxidation to C11 and C12 hydroxylation and C7 oxidation [95,96]. These findings highlight the importance of considering substrate orientation, pocket plasticity, and persistent near-attack conformations in P450 design.

Existing design cases in non-diterpenoid or general heme proteins also support this approach. Studies of flavonoid P450 pocket design, artificial heme enzymes, and NovoChrome collectively demonstrate that the distal pocket of heme proteins can be remodeled via computational design, ancestral sequence information, key pocket residue constraints, or experimental evolution, thereby altering substrate positioning and reaction selectivity [97,98,99]. For diterpenoid pathway-related P450s, this perspective, together with enzyme–substrate prediction rules, suggests that mutations should not be guided solely by conserved P450 motifs or overall sequence similarity. Instead, design should focus on the target diterpenoid skeleton, target carbon atoms for oxidation, heme–substrate distances, oxygen transfer angles, pocket hydrophobicity, and electron transfer compatibility.

The design logic for P450s has the potential to address several long-standing challenges in diterpenoid pathway reconstruction, including insufficient regioselectivity, uncontrolled sequential oxidation, difficulty in accumulating unstable intermediates, poor expression of plant P450s in microbial hosts, and incompatibility with heterologous CPRs or redox partners. Multiple cases of diterpenoid P450s have shown that these challenges do not occur in isolation. CYP720B1/PtAO can sequentially oxidize diterpene alcohol and diterpene aldehyde intermediates, demonstrating the multi-substrate and multi-step oxidative capacity of P450s in resin acid biosynthesis [100]. CYP88A can also catalyze consecutive oxidation steps from *ent*-kaurenoic acid to GA12 in gibberellin biosynthesis [101]. Such sequential oxidative capacity is advantageous for pathway compression, but in heterologous reconstruction it may also lead to excessive consumption of intermediates or make product distributions difficult to control. Taxadiene 5α-hydroxylase/CYP725A4 further illustrates that diterpenoid P450s, even when responsible for a defined pathway step, may generate multiple by-products, and the target hydroxylated product is not necessarily the major product [102]. Therefore, future design of diterpenoid P450s should aim not only to enhance catalytic activity, but also to restrict undesired binding poses, off-target oxidation sites, and overoxidation routes.

In particular, when a specific plant lineage is known to be enriched in a given diterpenoid skeleton and certain P450 subfamilies are expanded within that lineage, potential reaction templates can first be identified from natural candidate enzymes. Structural modeling, substrate docking, molecular dynamics, and generative pocket design can then be used to propose engineering strategies. In this way, lineage signals, natural enzyme discovery, and generative design can form a continuous framework. Plant evolution provides the candidate space, substrate–enzyme models provide a basis for prioritization, and structural design further expands or optimizes the available enzymatic functions.

In addition, chassis optimization [103,104,105] and the introduction of non-natural catalytic modules [106,107,108] will become important complements to diterpenoid pathway engineering. Plant P450s often face limitations in heterologous hosts, including membrane localization, electron transfer, and NADPH supply. Therefore, yeast platforms optimized for redox balance, plant transient expression systems, photosynthetic chassis, and cell-free systems may each play important roles in different contexts. Studies on artificial metalloenzymes and non-natural cofactors further suggest that the binding affinity between protein scaffolds and cofactors can be optimized, enabling abiotic catalysis in complex cytoplasmic environments and allowing the regulation of selectivity between competing reactions [106,107,108]. Although these non-natural catalytic systems do not directly mimic plant diterpenoid biosynthesis, they provide potential tools for late-stage diversification of diterpenoid skeletons and for transformations that are difficult to achieve using natural enzymes alone.

In the long term, Further work is needed to move plant diterpenoid biosynthesis research from “discovering individual missing enzymes” toward “predicting, designing, and reconstructing entire reaction networks.” Nevertheless, with the refinement of diterpenoid structure databases, the accumulation of enzyme functional data, and advances in protein structure prediction and generative models, research on plant-derived diterpenoid biosynthesis is expected to gradually shift from empirical discovery toward mechanism-driven prediction and design. The evidence-guided framework integrating scaffold enrichment with gene family evolution provides a starting point for such a closed-loop system. It first addresses where diterpenoid chemical innovation occurs during plant evolution and which enzyme families may have participated in this process. Subsequently, substrate–enzyme mapping and structural modeling can further determine which candidate enzymes are likely to catalyze specific reactions on particular diterpenoid skeletons. Finally, generative design and engineered chassis enable researchers to test, optimize, and even reshape these catalytic capabilities.

## Figures and Tables

**Table 1:** Summary of major strategies for discovering plant diterpenoid biosynthetic pathways.

Strategy	Main Evidence/Data	Typical Application	Strengths	Limitations	Refs.
Metabolic gene cluster mining	Genome assembly, gene proximity, collinearity, cluster prediction	Identification of physically clustered pathway genes	Effective when pathway genes are genomically clustered	Many plant pathway genes are dispersed; proximity does not prove function	[25,26]
Co-expression analysis	Transcriptomes across tissues, stages, or treatments	Screening genes co-expressed with known diTPSs or metabolite accumulation	Useful for non-clustered pathways; narrows candidate pools	Stress-responsive or tissue-specific genes may generate false positives	[17,18]
Homology-based prediction	BLAST/HMMER, known characterized enzymes	Identification of conserved diTPSs or related P450s	Simple and effective for conserved reaction types	Poor performance for novel reactions or highly diverged enzymes	[35,36]
Spatiotemporal expression association	Tissue-specific or inducible expression plus metabolite localization	Linking metabolite accumulation sites with candidate genes	Useful when metabolites accumulate in specific tissues or conditions	Expression overlap alone cannot establish biochemical function	[37]
Proteomics/ABPP	Protein abundance, substrate probes, activity-based labeling	Functional-level screening of substrate-binding enzymes	Closer to enzyme activity than transcriptomics	Probe design and membrane-protein detection are technically difficult	[38]

## Data Availability

All data generated and analyzed in this study are provided in the Supplementary Materials.

## Supplementary Materials

The following supporting information can be downloaded at: https://www.mdpi.com/article/10.3390/molecules31152653/s1. References [109,110,111,112,113,114,115,116,117,118,119,120,121,122,123,124,125,126,127,128,129,130,131,132,133,134,135,136,137,138,139,140,141,142,143,144,145,146,147,148,149,150,151,152,153,154,155,156,157,158,159,160,161,162,163,164,165,166,167,168,169,170,171,172,173,174,175,176,177,178,179,180,181,182,183,184,185,186,187,188,189,190,191,192,193,194,195,196,197,198,199,200,201,202,203,204,205,206,207,208,209,210,211,212,213,214,215,216,217,218,219] are included in the Supplementary Materials.

## Abbreviations

The following abbreviations are used in this manuscript:

diTPSs	Diterpene synthases
GGPP	Geranylgeranyl diphosphate
P450s	Cytochrome P450 monooxygenases
PSMs	Plant specialized metabolisms
17-HGL-DTGs	17-Hydroxygeranyllinalool diterpene glycosides
MGCs	Metabolic gene clusters
MeJA	Methyl jasmonate
UGTs	UDP-dependent glycosyltransferases
BAHD	BAHD acyltransferases
CPS	Copalyl diphosphate synthase
KSL	Kaurene synthase-like
ABPP	Activity-based protein profiling
GL	Geranyllinalool
Glc	Glucosyl
Rha	Rhamnosyl
Mal	Malonyl
TMTT	4,8,12-Trimethyltrideca-1,3,7,11-tetraene
*ent*-CPP	*ent*-Copalyl diphosphate
ADHs	Alcohol dehydrogenases
TXS	Taxadiene synthase
T5αOH	Taxane 5α-hydroxylase
TAT	Taxadiene-5α-ol-O-acetyl transferase
T10βOH	Taxane 10β-hydroxylase
T13αOH	Taxane 13α-hydroxylase
T2αOH	Taxane 2α-hydroxylase
T9αOH	Taxane 9α-hydroxylase
T7βOH	Taxane 7β-hydroxylase
T1βOH	Taxane 1β-hydroxylase
TBT	Taxane-2α-O-benzoyltransferase
DBAT	10-Deacetyl baccatin III-10-O-acetyltransferase
BAPT	Baccatin III-3-amino,13-phenylpropanoyltransferase
T2′αOH	Taxane 2′α-hydroxylase
DBTNBT	3′-N-Debenzoyl-2′-deoxytaxol-N-benzoyltransferase
PAM	Phenylalanine aminomutase
PCL	β-Phenylalanine coenzyme A ligase
MD	Molecular Dynamics
MM/PBSA	Molecular Mechanics/Poisson–Boltzmann Surface Area
